# Pyrimidone inhibitors targeting Chikungunya Virus nsP3 macrodomain by fragment-based drug design

**DOI:** 10.1371/journal.pone.0245013

**Published:** 2021-01-22

**Authors:** Sixue Zhang, Atefeh Garzan, Nicole Haese, Robert Bostwick, Yohanka Martinez-Gzegozewska, Lynn Rasmussen, Daniel N. Streblow, Mark T. Haise, Ashish K. Pathak, Corinne E. Augelli-Szafran, Mousheng Wu

**Affiliations:** 1 Drug Discovery Division, Chemistry Department, Southern Research, Birmingham, Alabama, United States of America; 2 Vaccine and Gene Therapy Institute, Oregon Health & Science University, Beaverton, Oregon, United States of America; 3 Drug Discovery Division, High-Throughput Screening Center, Southern Research, Birmingham, Alabama, United States of America; 4 Department of Genetics, University of North Carolina School of Medicine, Chapel Hill, North Carolina, United States of America; University of East Anglia, UNITED KINGDOM

## Abstract

The macrodomain of nsP3 (nsP3MD) is highly conserved among the alphaviruses and ADP-ribosylhydrolase activity of Chikungunya Virus (CHIKV) nsP3MD is critical for CHIKV viral replication and virulence. No small molecule drugs targeting CHIKV nsP3 have been identified to date. Here we report small fragments that bind to nsP3MD which were discovered by virtually screening a fragment library and X-ray crystallography. These identified fragments share a similar scaffold, 2-pyrimidone-4-carboxylic acid, and are specifically bound to the ADP-ribose binding site of nsP3MD. Among the fragments, 2-oxo-5,6-benzopyrimidine-4-carboxylic acid showed anti-CHIKV activity with an IC_50_ of 23 μM. Our fragment-based drug discovery approach provides valuable information to further develop a specific and potent nsP3 inhibitor of CHIKV viral replication based on the 2-pyrimidone-4-carboxylic acid scaffold. *In silico* studies suggest this pyrimidone scaffold could also bind to the macrodomains of other alphaviruses and coronaviruses and thus, have potential pan-antiviral activity.

## Introduction

Chikungunya Virus (CHIKV) is a re-emerging virus that is primarily transmitted by mosquitoes [1,2]. CHIKV belongs to the Alphavirus genus of the *Togaviridae* family which includes the “old world” and “new world” alphaviruses. The “old world” alphaviruses, including CHIKV, Sindbis Virus (SINV), Semliki Forest Virus (SFV) and O’nyong nyong virus (ONNV), usually cause fever, rashes and arthritis. The “new world” alphaviruses, including Venezuelan Equine Encephalitis Virus (VEEV) and Eastern Equine Encephalitis Virus (EEEV), tend to cause encephalitis [3–5]. The outbreaks of CHIKV infection spread from mainly tropical regions in Africa and Asia to other countries in Europe and the Americas [6–8]. CHIKV has become a substantial public health problem due to pan-epidemic infections, severe symptoms and reported fatal cases from acute infection [9,10]. The most common symptoms are fever and joint pain, and other symptoms include headache, rash, muscle pain and even neurological disorders [11–13]. Although CHIKV infections are rarely fatal, most patients develop a chronic disease, which is characterized most often by persistent and disabling polyarthritis [14–16]. Currently, there is no FDA-approved vaccine to prevent or drug to treat CHIKV infection.

CHIKV has approximately a 12kb positive-sense, single strand RNA (+ssRNA) genome that encodes four non-structural proteins (nsP1, nsP2, nsP3 and nsP4) and five structural proteins (C, E1, E2, E3 and 6K) [13,17]. All four nsP proteins in combination with host factors form the replication machinery tethered to cytoplasmic vacuoles and have distinct enzymatic activities responsible for viral RNA replication [18,19]. The nsP1 has methyl- and guanyl-transferase activities, which are important to add a cap structure to CHIKV genomic RNA. The RNA capping can effectively protect RNA from nuclease degradation, initiate protein translation and escape innate immunity [20,21]. The nsP2 has a helicase-domain at the *N*-terminus processing 5’-3’ RNA helicase and RNA annealing activities [22]. The *C*-terminus of nsP2 contains a papain-like protease domain, which is essential for the cleavage of precursor polyprotein P1234 into matured nsP1, nsP2, nsP3 and nsP4 proteins [23,24]. The nsP4 is an RNA-dependent RNA polymerase catalyzing the formation of negative-sense, genomic and subgenomic viral RNAs [25,26]. The nsP3 has a highly conserved macrodomain across species at the *N*-terminus, a central zinc-binding domain, and a *C*-terminal hypervariable domain (HVD). The zinc-binding domain and the hypervariable domain are required for the interactions with other nsPs and the host factors necessary for the replication complex formation and tethering to cytoplasmic vacuoles [27–30]. The *N*-terminal macrodomain has ADP-ribose 1”-phosphate phosphatase activity and it can bind to DNA, RNA, poly(ADP-ribose) (PAR) and ADP-ribose [29]. Recent work has demonstrated that CHIKV nsP3 macrodomain (nsP3MD) possesses mono(ADP-ribose) (MAR) hydrolase activity, which specifically removes the MAR moiety from aspartate and glutamate residues but not from lysine residues in substrate proteins [28]. Mutants affecting MAR hydrolase activity and ADP-ribose binding slow viral replication in mammalian cells and reduce CHIKV virulence in mice [28]. These results suggest that the nsP3 macrodomain is critical for CHIKV replication and may be a pharmaceutical target for anti-CHIKV drug discovery.

Fragment-based drug discovery (FBDD) is well-established in biopharmaceutical and biotechnology companies as an alternative and complementary approach to conventional high-throughput screening (HTS) to jump start a drug discovery program. Starting from fragment-based approaches, the B-Raf kinase inhibitor Vemurafenib and the Bcl-2 inhibitor Venetoclax were developed for the treatment for late stage melanoma and chronic lymphocytic leukemia, respectively [31]. Many FBDD-derived leads are also in varying stages of clinical trials [31]. FBDD can offer advantages over conventional structure-activity-relationship (SAR) approaches (e.g., HTS), such as less number of compounds required for the screening, a higher hit rate, more efficient binders and an easier path to chemical optimization [32]. FBDD approach involves the detection of low molecular weight chemical structures as starting points by screening small fragment libraries using biophysical assays, NMR, molecular docking or X-ray crystallography [33]. X-ray crystallography is commonly used for fragment-based screening to provide the structural information of protein-fragment interactions to guide the optimization of the fragment into a high-affinity lead by adding additional chemical groups to enhance binding or combining two fragments to generate improved binding small molecules. This can lead to synthesizing a fewer number of compounds than in traditional SAR approaches [32,33]. Strategies to grow the fragments into drug-like leads include fragment evolution, fragment linking, fragment self-assembly and fragment optimization [32]. Compared to other methods for fragment screenings, such as NMR and biophysical screening, the disadvantage of X-ray crystallography has been the relatively low throughput [31,33]. Through a decade of development in the pharmaceutical industry, techniques like compound cocktailing of fragment library, streamline data collection, automated data processing and ligand fitting have transformed X-ray crystallography into a highly efficient technique that is suitable for fragment screening [34,35].

Inhibitors have been developed by traditional SAR or from the conventional HTS specifically for CHIKV nsP1, nsP2 and nsP4 [36]. So far, there are no reported inhibitors targeting alphavirus nsP3 or nsP3MD. In this study, we identified small fragments via a fragment-based approach to inhibit the CHIKV viral replication. All fragments discovered by X-ray crystallography were shown to localize at the distal ribose binding site of ADP-ribose in the nsP3MD crystal structure and share a very unique chemistry scaffold, 2-pyrimidone-4-carboxylic acid. One of the fragments from our compound library, SRI-43750, showed anti-CHIKV-activity with an IC_50_ of 23 μM. Our structural biology and *in silico* studies provide valuable information for medicinal chemists to further develop nsP3-specific inhibitors targeting the CHIKV viral replication mechanism using the unique chemical scaffold described herein. Moreover, additional *in silico* docking studies suggest this scaffold could potentially bind to the nsP3MD of other alphaviruses and coronaviruses including SARS-CoV-2, indicating that the pyrimidone inhibitors can also have pan-antiviral activity.

## Materials and methods

### Chemicals

All fragment compounds were purchased from Life Chemicals, Enamine, LabNetwork, AmBeed, ChemDiv, Combi-blocks and Chembridge commercial sources. Methyl 2-oxo-1*H*-quinazoline-4-carboxylate (SRI-44019) was synthesized in-house by esterification of 2-oxo-1*H*-quinazoline-4-carboxylic acid in the presence of methanol. All purchased compounds were analyzed by liquid chromatography-mass spectrometry (LC-MS, Agilent) and high performance liquid chromatography (HPLC, Waters). The purity of all compounds reported is > 95%. SRI-43750 was also analyzed by ^1^HNMR (Agilent at 400 MHz) and the purity was 96% by HPLC. ADP-ribose was purchased from Sigma-Millipore. All reagents for molecular biology were purchased from Fisher Scientific. Crystallization materials and reagents were purchased from Hampton Research.

### Virtual screening and computational docking

Virtual screening (VS) and the follow-up docking studies were performed using Schrödinger Small Molecule Drug Discovery Suite [37]. A fragment compound library (molecular weight < 300, in total ~14K compounds) assembled from vendors such as Enamine, ChemBridge, and ChemDiv were virtually screened. The VS protocol implemented in Schrödinger [38,39] was used to screen the fragment library against the ADP-ribose binding site of nsP3MD crystal structure determined in the structural biology laboratory (PDB ID: 6VUQ). Using the docking score of adenosine (MW = 267) as the reference, a VS score of -7.5 kcal/mol was set as the cut-off value to select VS hits. For follow-up docking studies, the induced-fit docking protocol implemented in Schrödinger [40] was used to dock fragments to the ADP-ribose binding site of CHIKV nsP3MD as well as the ADP-ribose binding site of nsP3MD crystal structures of VEEV (PDB ID: 3GQO), MERS-CoV (PDB ID: 5HOL), SARS-CoV (PDB ID: 2FAV), and SARS-CoV-2 (PDB ID: 6W02).

### Expression and purification of nsP3MD

The nsP3 macrodomain (residues 1–160) containing a 6-histidine tag at the *N*-terminus was amplified from a plasmid containing full-length nsP3 (pcDNA-DEST47-nsP3) by PCR using the forward primer (CGCGGATCCCACCATCACCATCACCATGCACCGTCGTACCGGGTAAAAC) and the reverse primer (CGCCTCGAGTTAGGTCCGCATCTGTATGGCCTCAG) (Integrated DNA Technologies, BamHI and XhoI restriction sites are underlined). The PCR product was purified, digested and ligated into modified pGEX-4T-3 vector (Cytiva life sciences) in which the thrombin cleavage site was replaced with the TEV protease cleavage site. The final construct termed pGEX-nsP3MD was transfected into BL-21 Rosetta (DE3) pLysS *E*. *coli* competent cells (Millipore-Sigma, Catalog # 79056) for protein expression. A single colony was inoculated in Luria-Bertani (LB) broth medium and cultured at 37°C overnight. A 10 mL overnight culture was added to 500 mL of auto-induction medium [41] and cells were cultured at 37°C for approximately five hours. The temperature was lowered to 18°C and cells were further cultured overnight. Cells were harvested and resuspended in lysis buffer (20 mM Tris pH 8.0, 300 mM NaCl, pH 8.0) with 1 mg/mL lysozyme (Millipore-Sigma) and 1 pellet of SIGMAFAST protease inhibitor (Millipore-Sigma). Cells were lysed using sonication and cell pellets were spun down at 15,000 rpm for one hour. The supernatant was loaded onto a glutathione-sepharose column (Cytiva life sciences). The column was washed with lysis buffer and GST-nsP3MD protein was eluted in 25 mM reduced glutathione (Millipore-Sigma) prepared in lysis buffer. GST-fused TEV protease, with a thrombin cleavage site between GST and TEV protease prepared in the lab, was added into the protein elution with a ratio of 1:20 (w/w). The protein mixture was passed through a G-25 desalting column (Cytiva life sciences) and incubated at 4°C overnight. The protein solution was reloaded onto a glutathione-sepharose column to remove GST and GST-TEV. The nsP3MD was further purified by size-exclusion chromatography using a Superdex-75 column (16/600, Cytiva life sciences) with gel filtration buffer (20 mM Tris pH7.5, 150 mM NaCl). The nsP3MD protein was concentrated to 18 mg/mL and stored at -80°C.

### Crystallization, compound soaking, data collection and structural determination

The nsP3MD was crystallized at 18°C by mixing 1.5 μL nsP3MD protein with 1.5 μL reservoir solution (21–24% PEG 400, 0.1 M sodium citrate pH5.6, 10% isopropanol) using the sitting-drop vapor diffusion method. ADP-ribose was prepared in water and all fragments were prepared in DMSO at a concentration of 500 mM. The nsP3MD crystals were washed in crystal cryo-protectant buffer (42% PEG 400, 0.1M sodium citrate pH5.6) and soaked at 18°C overnight with 25 mM ADP-ribose or with fragments prepared in the cryo-protectant buffer. The nsP3MD crystals were directly frozen in liquid N_2_ for storage. Most of the X-ray diffraction data were collected at SER-CAT (Southeast Regional Collaborative Access Team) beamline ID-22 at Advanced Proton Sources (Argonne National Laboratory, Illinois, and USA). Most data were processed by XDS [42] and scaled by Scala [43]. Some of the X-ray diffraction data were collected at home-source Rigaku R-AXIS IV++ with a Pilatus 200K detector (Rigaku Corporation) and processed with HKL3000 [44]. All protein structures were determined by molecular replacement using Phaser-MR program [45]. The nsP3MD monomeric structure from an nsP3MD structure (PDB ID: 3GPG) [29] was used as a search model. Structures were refined by Refmac 5 [46] and edited in COOT [47]. After the final nsP3MD models with water molecules were refined, fo-fc maps [47] were examined to check the extra electron density for the bound fragments in the ADP-ribose binding site. All molecular models of fragments were fit into the electron density and nsP3-fragment models were refined to an ideal geometry checked by Procheck [46,48]. The final models were deposited in the Protein Data Bank (www.rcsb.org). All statistics for data collection and model refinement are listed in S1 Table (Supplementary Information).

### Thermal shift assay

Binding of SRI-43750 to the CHIKV nsP3 MD was detected using the GloMelt thermal shift protein stabilization kit (Biotium) according to the manufacturer instruction. The CHIKV nsP3MD (1.7 μg/uL) was mixed with either ADP-ribose at 2mM or SRI-43750 at 0.1, 1, or 5 μM with 0.05% final DMSO concentration and 1x GloMelt Dye (Biotium) in a final volume of 20 μL of assay buffer (100mM Tris 300mM NaCl, pH7.5). IgG (Biotium) and nsP3MD protein solution alone in the assay buffer were set up as controls. The mixtures were added to a Quantstudio 7 thermal cycler (Invitrogen) and the denaturation of the CHIKV nsP3MD protein was measured during a melt curve ranging from 25-95°C by 0.3°C steps. Fluorescence emitted by binding of the dye to exposed hydrophobic regions of the protein during the melt curve was used to calculate the melting temperature (T_m_) from the mid-log transition on a Boltzman model (GraphPadPrism 9).

### Anti-CHIKV cellular assay

Normal human foreskin fibroblasts (ATCC # CRL-2522) were modified by knocking out IRF3 (transcription factor regulator of type I interferon induction) and stably transfecting them with telomerase (THF-Neo-ΔIRF3). Cells were grown in DMEM supplemented with 2% FBS, 1% Penicillin/Streptomycin, 1% HEPES, 1% NEAA and 1% sodium pyruvate. For the assay, cells were harvested, suspended at 150,000 cells/mL, and seeded in 384-well black, clear bottom plates (Corning, Catalog # 3764BC) at 3,000 cells/well in 20 μL of the cell suspension. The assay plate wells were pre-drugged with 5 μL of media (columns 1,2,23,24) or media containing test compounds diluted to 6x final concentration (columns 3–22). Since the test compound stock solutions were in 100% DMSO, the final assay concentration of DMSO in each well was maintained at 0.4%. After overnight incubation at 37°C/5% CO_2_, the wells in columns 3–24 were inoculated at approximately 0.01 MOI (Multiplicity of infection) with Chikungunya strain 181/25 nanoLuc reporter virus by adding 5 μL of 6X final virus dilution in media. Media only (5 μL) was added to the wells in columns 1 & 2. The 32 wells in columns 1 & 2 (cells only) and the 32 wells in columns 23 & 24 (cells plus virus, no compound) were used for low and high signal controls, respectively. Plates were returned to the incubator for an additional 48 hours after which 30 μL Nano-Glo reagent (Promega, Catalog # N1110) were added to each well. After incubating for 10 minutes at room temperature, luminescence was read using a PHERAstar FSX microplate reader (BMG LABTECH GmbH). The raw signal data for each well was normalized to % inhibition by the following formula:

%inhibition=100*[(testcompound‐avghighsignal)/(avglowsignal−avghighsignal)].

IC_50_ (the half maximal inhibitory concentration) values were calculated by a four-parameter logistic fit of the concentration response data using ActivityBase XEDesigner Fit formula #205.

### Cellular cytotoxicity assay

To assess cytotoxic effect of compounds, a cell viability assay was performed. Cells were suspended in DMEM supplemented with 2% FBS, 1% Pen/Strep, 1% Hepes, 1% NEAA and 1% Sodium Pyruvate were seeded in 384-well black, clear bottom plates (Corning, Catalog # 3764BC) at 3,000 cells/well by adding 20 μL of the cell suspension. The assay plate wells were pre-drugged with 5 μL of media (columns 1 and 2), 5 μL of media containing test compounds diluted to 6x final concentration (columns 3–22), or 5 μL of media containing 600 μM hyamine hydroxide (columns 23 & 24), maintaining 0.4% DMSO in all wells. Wells in columns 1 & 2 were the high signal controls (100% viability) and wells in columns 23 & 24 were the low signal controls (0% viability). After overnight incubation at 37°C/5% CO_2_, 5 μL of media was added to each well to bring the final volume to 30 μL and the plates were returned to the incubator for 48 h. Cell Titer Glo (30 μL) was then added to the wells and luminescence was read using a PHERAstar FSX microplate reader (BMG LABTECH GmbH). The raw signal data for each well was normalized to % viability by the following formula:

%viability=100*[(testcompound‐avglowsignal)/(avghighsignal−avglowsignal)].

CC_50_ (The 50% cytotoxic concentration) values were calculated by a four-parameter logistic fit of the concentration response data using ActivityBase XEDesigner Fit formula #205.

## Results and discussion

### Optimization of crystal soaking for nsP3MD crystals

nsP3MD crystals were found to be very robust and stable in different conditions during the crystallization, suggesting that nsP3MD crystals are suitable for fragment-based screening using crystal soaking. Moreover, we were focusing on identifying fragments which can bind to the ADP-ribose binding site since studies have shown that the binding site is very critical for CHIKV viral replication [28]. In the crystal structure [29], the ADP-ribose binding site was not involved in crystal contacts. All of these observations suggest that it is very promising to find a fragment or inhibitors of nsP3MD by using X-ray crystallography as an approach in the fragment-based screening. To improve the throughput of crystal soaking, it is advantageous to prepare the compound in a cryo-protectant buffer for crystal soaking to avoid further manipulation at the crystal freezing stage [33]. In this study, we tried to optimize a simple cryo-protectant buffer in which the crystals were very stable after soaking with fragments overnight and the crystals were then directly frozen in liquid nitrogen for data collection. For this purpose, ADP-ribose was used to optimize the protocol since the high-resolution co-crystal structure of nsP3MD-ADP-ribose was determined prior to the attempt of co-crystallization with a fragment [29].

**Fig 1:**
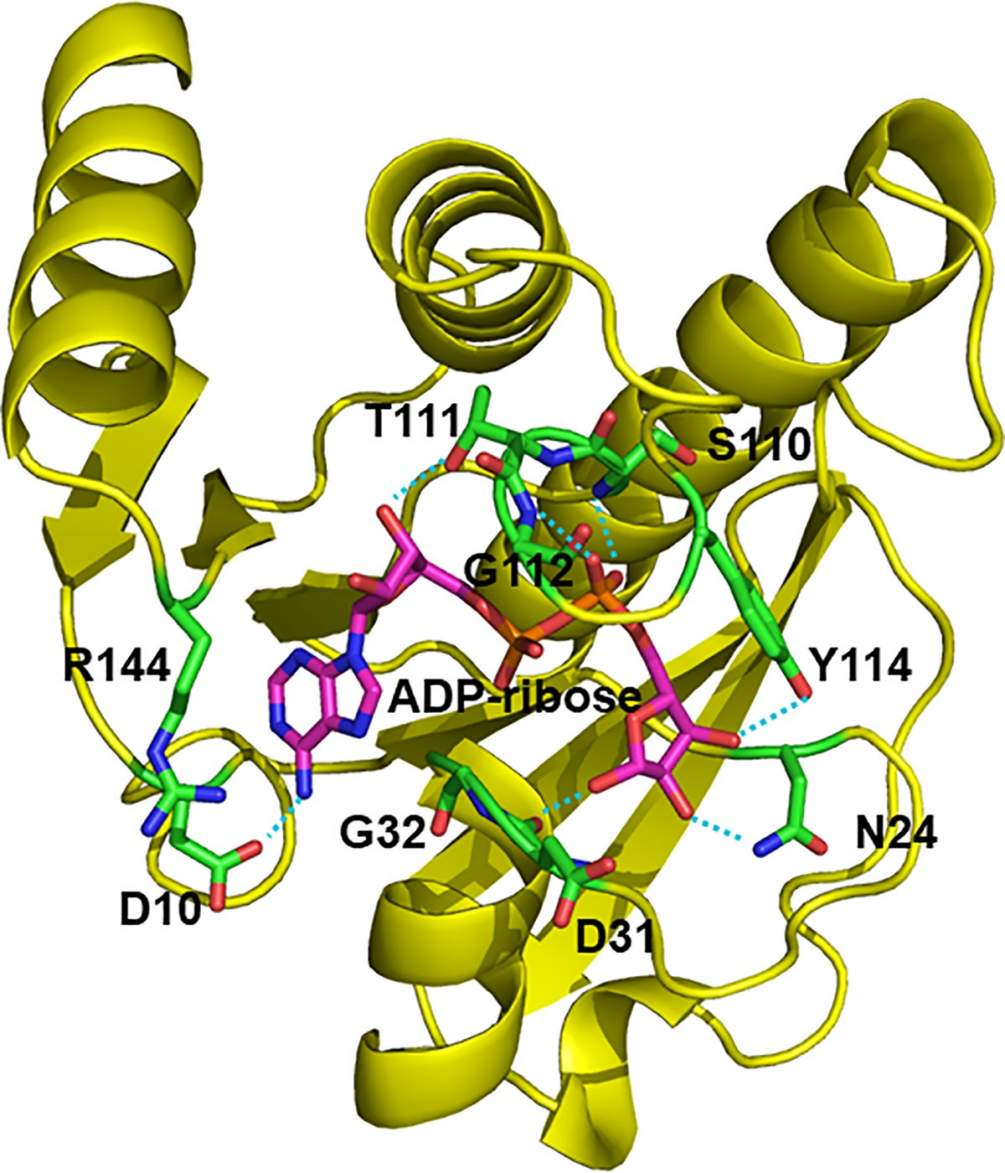
The co-crystal structure of CHIKV nsP3MD with ADP-ribose. CHIKV nsP3MD structure is shown in yellow cartoon. ADP-ribose is shown in magenta sticks in the center of the cavity. Residues of nsP3MD that interact with ADP-ribose are shown in green sticks. The hydrogen bonds between nsP3MD and ADP-ribose are shown in cyan dashed lines. All graphic presentations for molecular structures were generated using Pymol [49].

**Fig 2:**
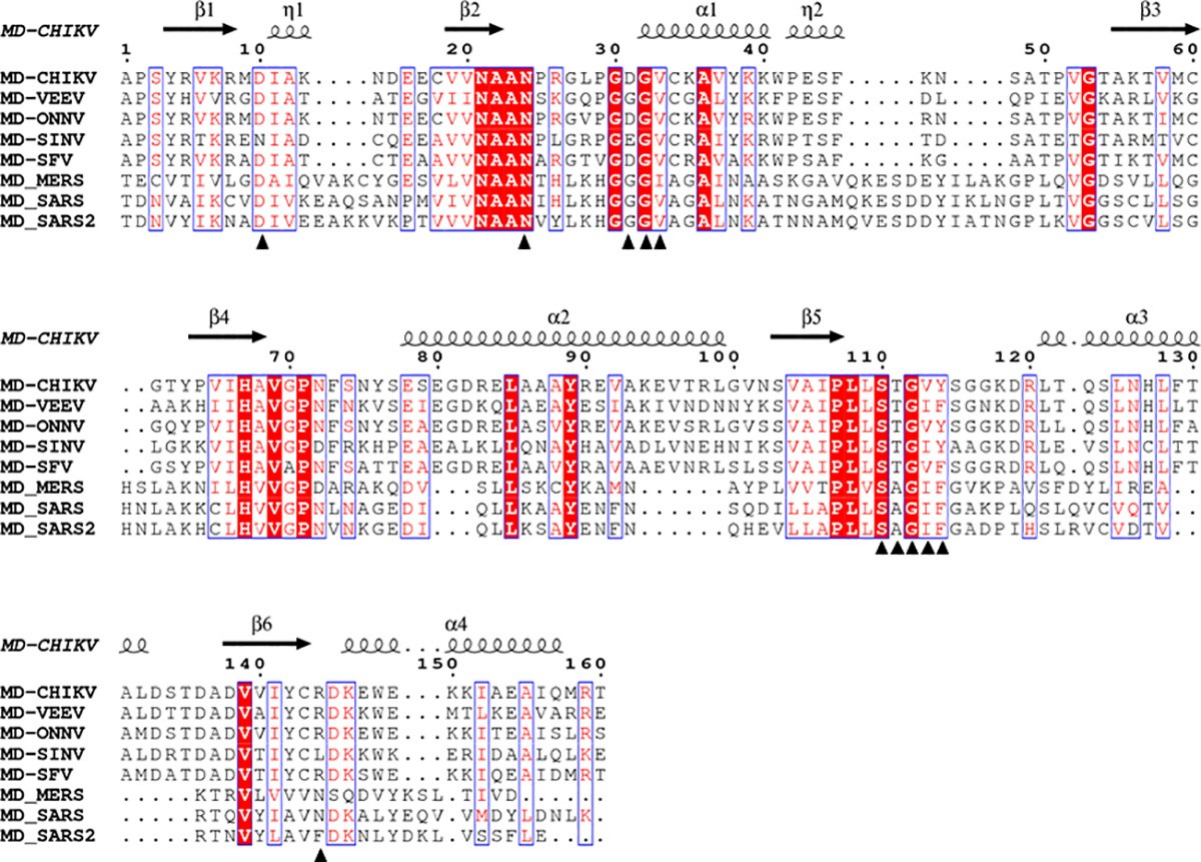
Sequence alignment of alphavirus and coronavirus nsP3 macrodomains (MD). Residue numbers above the sequence correspond to CHIKV nsP3 macrodomain (UniProtKB: Q5XXP4). The secondary structure from the co-crystal structure of nsP3MD with ADP-ribose is shown on the top of the alignment. Residues interacting with ADP-ribose are highlighted in black triangles. Other protein sequences shown here include macrodomains from VEEV (UniProtKB: P36328), ONNV (UniProtKB: P13886), SINV (UniProtKB: P03317), SFV (UniProtKB: P08411), SARS-CoV (PDB ID: 2FAV), MERS-CoV (PDB ID: 5HOL) and SARS-CoV-2 (SARS2) (PDB ID: 6W02). This figure was generated by using ESPript 3.0 [50].

Native nsP3MD crystals were soaked with ADP-ribose in three different cryo-protectant buffers (cryo-buffers): (i) a cryo-buffer containing glycerol and iso-propanol (26% PEG400, 0.1*M* sodium citrate, pH5.6, 10% 2-propanol, 25% glycerol), (ii) a cryo-buffer containing glycerol but no isopropanol (26% PEG400, 0.1*M* sodium citrate, pH 5.6, 25% glycerol), and (iii) a cryo-buffer without glycerol and iso-propanol (42% PEG400, 0.1*M* sodium citrate, pH5.6). The crystals were directly frozen in the liquid nitrogen for X-ray diffraction data collection. Three structures of nsP3MD-ADP-ribose were determined and found to be very stable after overnight soaking in all three cryo-buffers. The electron density of ADP-ribose was clearly observed in all three structures. The results also suggest that the volatile organic solvent, *iso*-propanol, is not a critical component for crystal stability. Therefore, the simplest cryo-buffer, 42% PEG400, 0.1*M* sodium citrate pH 5.6 without isopropanol, was chosen as the cryo-buffer to soak fragments from the virtual screen. It significantly improved the crystal handling and efficiency of crystal soaking. The crystal structure of nsP3MD-ADP-ribose (Fig 1) was found to be identical to the reported structure (PDB: 3GPO) [29]. In the structure, residues D10, G32, V33 and R144 are responsible for the specific recognition of adenine nucleobase. The T111 provides a hydrogen bond with the adenine proximal ribose. The main chain NH groups of residues S110, G112, and Y114 are involved in the phosphate binding via hydrogen bonds. Residues Y114, D31 and N24 make hydrogen bonds with the OH group on the distal ribose. Notably, most residue contacts with ADP-ribose are highly conserved within the alphavirus family (Fig 2).

### Virtual screening

Virtual screen using computational molecular docking against the crystal structure of CHIKV nsp3MD solved in our laboratory (PDB ID: 6VUQ) was performed to identify hits that can bind to the viral protein and potentially inhibit CHIKV viral replication. A total of approximately 14K fragment compounds (defined as MW < 300) were virtually screened against the ADP-ribose binding site of the CHIKV nsp3MD molecular structure. Based on docking scores, virtual binding modes, and drug-like properties (e.g., solubility > 10 μM, no reactive groups through PAINS [51] filtration), 40 compounds (S2 Table, Supplementary Information) were selected and purchased for the first round of a crystal soaking screen.

### Fragment screening against nsP3MD by X-ray crystallography

**Fig 3:**
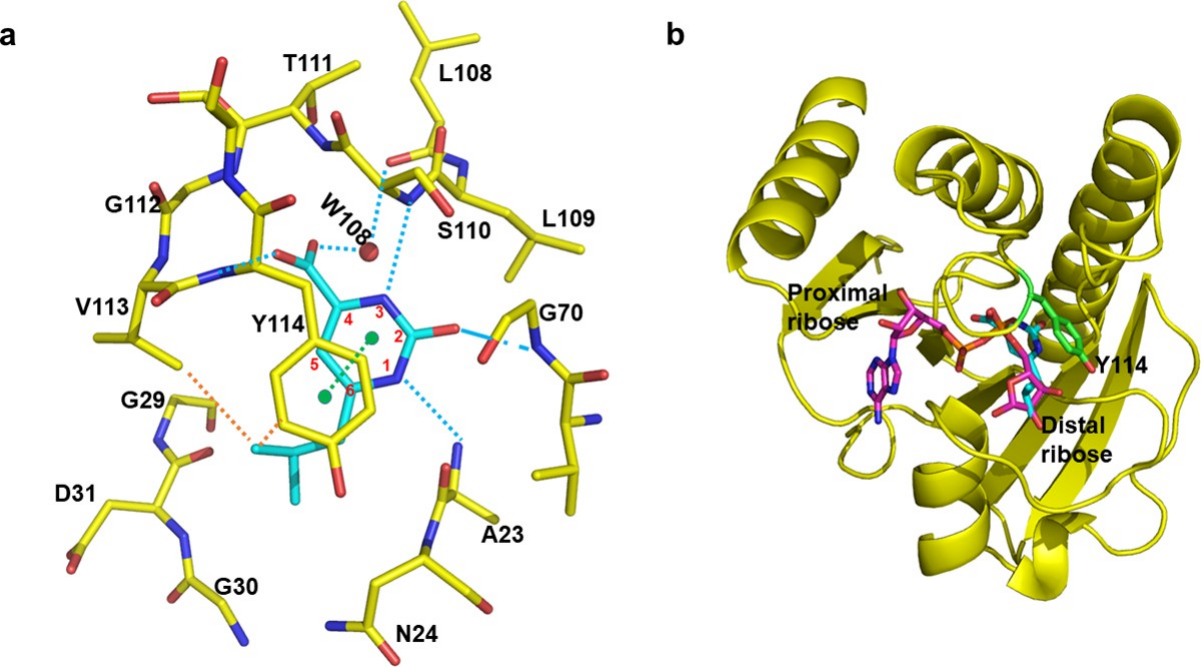
The co-crystal structure of CHIKV nsP3MD with SRI-40582. a) The interaction network between nsP3MD and SRI-40582. SRI-40582 is colored in cyan sticks. Key residues of nsP3 that interact with SRI-40582 are highlighted in yellow sticks. Hydrogen bonds, π-π stacking, and hydrophobic contacts are indicated by cyan, green, and orange dashed lines, respectively. Pyrimidine numberings are shown in red around the ring structure. b) Comparison of SRI-40582 and ADP-ribose in the nsP3MD structure. The nsP3MD structure is shown in yellow cartoon. A SRI-40582 molecule is colored in cyan sticks and ADP-ribose in magenta sticks. The Y114 of nsP3MD is shown in green sticks.

Of the 40 fragments that were soaked in nsP3MD crystals, one fragment, 6-isobutyl-2-oxo-pyrimidine-4-carboxylate (SRI-40582), was found in the ADP-ribose binding pocket. In the co-crystal structure, SRI-40582 forms a π-π interaction, a hydrophobic interaction and a very extensive hydrogen-bonding network with nsP3MD as shown in Fig 3A. The pyrimidine ring of SRI-40582 forms a π-π interaction with the phenol group of Y114. The *iso*-butyl group of SRI-40582 contributes to the hydrophobic interaction with the phenol ring of Y114 and the side chain of V113. In addition to these interactions, an extensive hydrogen bonding network significantly contributes to the binding of SRI-40582 to nsP3MD (Fig 3A). The NH group at the 1-position of pyrimidine ring forms a hydrogen bond with the main chain amide group of A23. The oxo group at the 2-position of the pyrimidine ring makes a hydrogen bond with the main chain amide group of G70. The nitrogen at the 3-position of the pyrimidine ring makes a hydrogen bond with the main chain amide group of S110. The carbonyl group of carboxylic acid at the 4-position of SRI-40582 makes a hydrogen bond with the main chain amide group from G112 and Y114, while a water molecule bridges the hydrogen bond interaction between the hydroxyl group of carboxylic acid and carbonyl group from L108 (Fig 3A). Compared to the co-crystal structure of nsP3MD-ADP-ribose, 2-pyrimidone-4-carboxylic acid of SRI-40582 partially overlaps with the *beta* phosphate group of ADP and the *iso*-butyl group of SRI-40582 partially occupies the position of distal ribose of ADP-ribose (Fig 3B). This suggests that SRI-40582 could interfere with ADP-ribose binding to nsP3 in the cell.

### 2-Pyrimidone-4-carboxylic acid is the minimal scaffold for binding to nsP3MD

In the co-crystal structure of SRI-40582 with nsP3MD, all three components of SRI-40582 (the *iso*-butyl group, pyrimidone ring and carboxylic acid group) made contact with nsP3MD. Thus, we investigated whether all three components were critical for SRI-40582 for binding to nsP3MD. Additional commercial compounds discussed below were soaked in nsP3MD crystals to address whether the *iso*-butyl group and carboxylic acid group can be removed or replaced, and whether the pyrimidone ring structure is critical for the binding in the co-crystal structure. Through these investigations we were able to identify the minimal structural requirement for binding to the nsP3MD structure.

**Fig 4:**
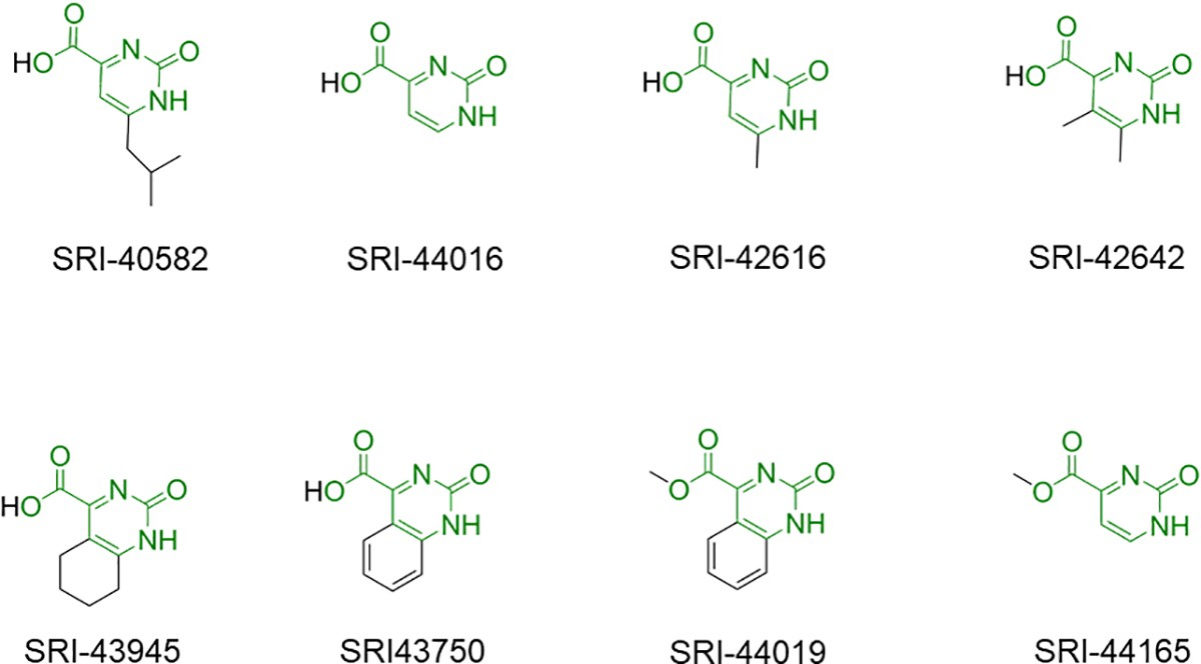
All fragments that formed co-crystal structures with nsP3MD. The common core structure is colored green with additional modifications depicted in black.

From the co-crystal structure of SRI-40582 in nsP3MD, the *iso*-butyl group of SRI-40582 contacts with V113 and Y114 (Fig 3A). The hydrophobic *iso*-butyl group stays in a hydrophilic pocket where the distal ribose moiety of ADP-ribose binds (Fig 1). To investigate whether the fragment lacking the *iso*-butyl group can still bind to the pocket, a fragment molecule without the *iso*-butyl group, 2-pyrimidone-4-carboxylic acid (SRI-44016) (Fig 4) was soaked into nsP3MD crystals, and the fragment was found in the nsP3MD co-structure. It is not surprising that the location of SRI-44016 in the crystal structure is the same as that of SRI-40582 (Fig 3) as both share the same π-π interaction and extensive hydrogen-bond network (Fig 3A). The structure of nsP3MD bound with SRI-44016 (Fig 4) suggests that the core, 2-pyrimidone-4-carboxylic acid, is sufficient for the binding to the nsP3MD structure and without the *iso*-butyl group in SRI-40582 the fragment can still bind to nsP3MD.

**Fig 5:**
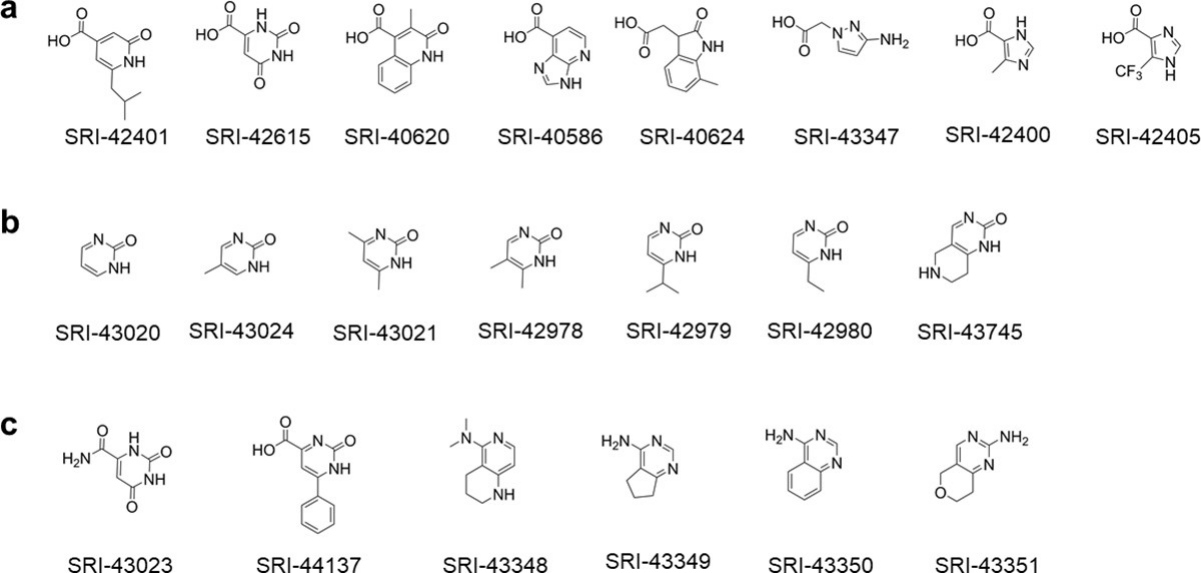
Fragments that failed to co-crystalize with nsP3MD. Shown are compounds that contain: a) a carboxylic acid group and other ring structures; b) a pyrimidone ring without carboxylic acid; and c) similar ring structures to the pyrimidone ring and with different chemical group substitutions on the ring structures.

To further investigate the importance of the 2-pyrimidone ring in the binding of SRI-44016 to nsP3MD, several commercial compounds containing a carboxylate group on different ring structures were used to explore whether the change on the pyrimidine ring or the replacement of pyrimidine ring with other ring structures can affect the binding to nsP3MD (Fig 5A). None of the compounds (Fig 5A) were found in the co-crystal structures. Compared to SRI-40582 in the co-crystal structure with nsP3MD (Fig 3A), one of these fragments, SRI-42401, could only lose one hydrogen bond contributed by the 3-position on the pyrimidine ring from the nsP3MD-SRI-40582 structural model (Fig 3A). The loss of this hydrogen bonding resulted in the failure to obtain the co-crystal structure of SRI-42401 with nsP3MD. This result suggests that the pyrimidone ring structure is critical for the fragments to bind to nsP3MD. For another fragment, SRI-42615, the NH-CO-NH triad on the pyrimidone ring is similar to that of SRI-44016 and SRI-40582; however, the fragment was not observed in the co-crystal structure. This suggests that the nitrogen atom at the 1-position on the pyrimidine ring should be a hydrogen donor while the nitrogen atom at the 3-position on the ring should be a hydrogen acceptor. Therefore, the proper triad structure on the pyrimidone ring for the binding should be NH-CO-N not NH-CO-NH or N-CO-NH.

To further investigate whether the carboxylic acid group at 4-position of the pyrimidone ring is essential for binding to nsP3MD, another set of compounds in which the 2-pyrimidone ring remained were soaked in nsP3MD crystals (Fig 5B). All fragments possess the same NH-CO-N triad on the pyrimidone ring but lack the carboxylic group on the pyrimidone ring compared to SRI-44016. However, none of these fragments formed nsP3MD co-crystal structures. As indicated in co-crystal structures described above, the carboxylic acid group forms multiple strong hydrogen bonds with nearby protein backbone or water molecules (Fig 3A). Thus, the contacts made by the carboxylic acid group contribute significantly to the overall binding for such small fragments in the nsP3MD crystal structure and also aid in the orientation of the ligands upon binding. This suggests that the carboxylic acid group is critical for small fragments such as SRI-40582 and SRI-44016 to bind to nsP3MD.

In addition to the studies above, more compounds with a similar structure to 2-pyrimidone-4-carboxylic acid (Fig 5C) were tested in this study. These compounds did not form any co-crystal structures with nsP3MD, which indicates that any changes to the carboxylic acid group or the pyrimidone ring skeleton would restrict the fragment binding to nsP3MD. Hence, it is concluded from all studies that 2-pyrimidone-4-carboxylic acid (SRI-44016) is the minimal scaffold for the binding of fragments to the nsP3MD crystal structure and both the pyrimidone ring and carboxylic acid group are critical for the binding. The fragments that bind to nsP3MD need to possess a very unique feature, such as the triad structure on the pyrimidone ring should be an NH-CO-N and the carboxylic acid should be on the 4-position on the pyrimidone ring.

### Exploring fragment growth based on the pyrimidone carboxylic acid scaffold

**Fig 6:**
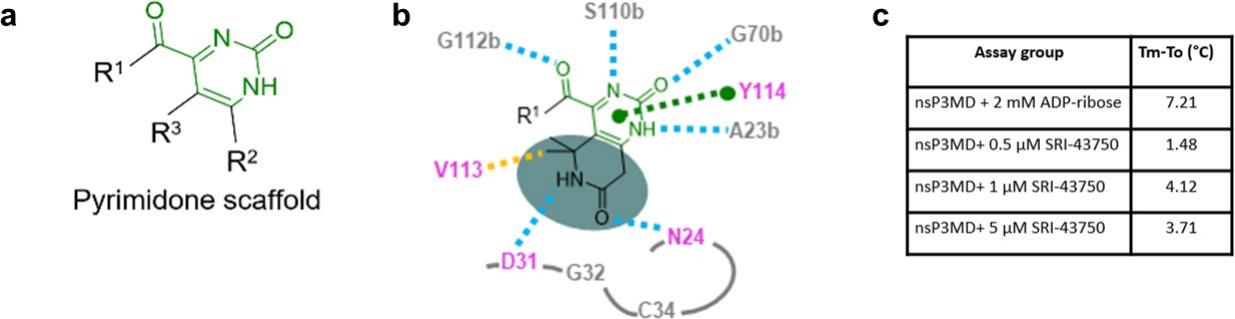
Fragment-based *in silico* design and thermal shift assay. a) Definition of the fragment scaffold. b) An ideal compound of R^2^ and R^3^ according to receptor-structure-based design. The residues on nsP3MD interacting with the compound are highlighted. The hydrogen bonds are labelled with cyan dashed lines. The π-π interaction is highlighted with a green dashed line. The hydrophobic interaction is highlighted with an orange dashed line. Residues which are highlighted in purple are critical for CHIKV viral replication. c) Thermal shift assay of nsP3MD with ADP-ribose and SRI-43750. The *T*_*m*_ is the melting temperature in the presence of ADP-ribose and SRI-43750 while *T*_*0*_ is the melting temperature of nsP3MD alone.

Next, fragment evolution [32] was explored as a strategy to expand the minimal fragment, 2-pyrimidone-4-carboxylic acid, to a drug-like molecule. The pyrimidone-carboxylate drug-like molecule for future analog development is exemplified in Fig 6A, in which R^1^, R^2^ and R^3^ represented different chemical groups attached to the pyrimidone ring. The fragment can be extended on R^2^ or R^3^ positions toward the distal ribose binding pocket to fill the space formed by the loop regions (residues 29–31) and N24 on nsP3MD as compared to the ADP-ribose in the binding site (Fig 3B). For example, different chemical groups (R^2^ and R^3^, Fig 6) such as methyl groups (SRI-42616 and SRI-42642, Fig 4) can be placed on 5- and 6-position on the pyrimidone ring.

To investigate the possibility of substitution on the pyrimidone ring, commercial compounds with different chemical groups or ring structures on 5- and 6- (R^3^ and R^2^) positions, SRI-42616, SRI-42642, SRI-43945 and SRI-43750 (Fig 4), were soaked into nsP3MD crystals. All four compounds were found in the co-crystal structures with nsP3MD. Similar to the *iso*-butyl group in SRI-40582, the π-π interaction and the hydrogen bonding network were not disturbed by the methyl group on the 6-position (SRI-42616) and dimethyl substitutions on both the 5- and 6-positions of the pyrimidone ring (SRI-42642). Hence, these two compounds were found in co-crystal structures with nsP3MD. Similarly, the two quinazoline compounds, 2-oxo-5,6,7,8-tetrahydro-quinazoline-4-carboxylic acid (SRI-43945) and 2-oxo-quinazoline-4-carboxylic acid (SRI-43750), have the closed ring structures at 5- and 6-position of the pyrimidine ring (Fig 4) and the extra rings also enhance the π-π interaction with the phenol ring of Y114 (Fig 3A) resulting in the binding to nsP3MD in the crystal structure. The results from these four fragments suggests that the pyrimidone ring can be developed on 5- and 6-positions of the pyrimidone ring in the future with other chemical groups to enhance the binding to the nsP3MD. Based on *in silico* modelling, an ideal compound, dimethylpiperidone pyrimidone compound (Fig 6B), was designed to fit into the ribose binding pocket of nsP3MD to interact with residue N24 and the loop region of residues 29–31 on nsP3MD. The target compound contains the 2-pyrimidone-4-carboxylate backbone, which can maintain the hydrogen-bonding network and π-π interaction. The extra ketone group on the piperidone ring structure can form an extra hydrogen-bond with residue N24 on nsP3MD. The dimethyl group on the piperidone ring can interact with nsP3MD V113 via hydrophobic interactions and the amide group can form a hydrogen bond with the side chain of D31 on nsP3MD (Fig 6B). Interestingly, unlike other conserved residues that interact with the pyrimidone scaffold (Fig 2), D31 is a variant residue. Therefore, a chemical moiety can be attached to pyrimidine scaffold to interact with D31 to increase the selectivity to CHIKV nsP3MD if needed.

On the other side of the pyrimidone molecule, different linkers or aliphatic chemical moieties on R^1^, such as SRI-44019 and SRI-44165 (Fig 4) can be coupled to the carboxylic acid to further extend the molecule into the AMP binding site (Fig 3B). Compounds SRI-44019 and SRI-44165, the methyl carboxylates of SRI-44016 and SRI-43750, respectively (Fig 4), were co-crystalized with nsP3MD. The co-crystal structures of these compounds are identical to their parent compounds except that the methyl group for both fragments are not visible due to the highly flexible orientation of the methyl group. These studies demonstrate that different analogs can also be developed in future with various chemical groups the R^1^-position (Fig 6A) to enhance the contact with the AMP binding pocket resulting in a potent specific drug-like pyrimidone lead compound.

All eight potential pyrimidone compounds discussed (Fig 4) were tested in the anti-CHIKV assay using normal human foreskin fibroblasts (NHDF) cells infected with Chikungunya nanoLuc reporter virus. Only SRI-43750 showed the inhibition of viral replication with an IC_50_ value of 23 μM, while all other compounds showed minimum activity with IC_50_ values > 40 μM. All eight compounds showed no cytotoxicity (CC50) to NHDF cells up to 40 μM concentration. These data suggest that the quinazoline ring of SRI-43750 with an extra phenyl ring attached to the pyrimidone ring enhances the π-π interaction and improves the antiviral activity. The binding of SRI-43750 to nsP3MD was also detected by thermal shift assay, in which the melt temperature increase (*Tm*-*To*) was about 4°C in the presence of 1–5 μM SRI-43750 (Fig 6C) while the *T*_*m*_ increase of nsP3MD was about 7°C in presence of ADP-ribose. This suggests that SRI-43750 can increase nsP3MD protein stability in solution similar to ADP-ribose. The co-crystal structure of nsP3MD with SRI-43750 and the results from thermal shift assay suggest SRI-43750 can bind to nsP3MD. However, compared to the ADP-ribose in the binding site of nsP3MD, SRI-43750 binding is only partially overlapped with that for the distal ribose moiety of ADP-ribose in the nsP3MD binding site (Fig 3B). Therefore, the binding affinity of SRI-43750 is likely not high enough to effectively inhibit the ADP-ribosylhydrolase activity of nsP3MD. In CHIKV-infected cells, nsP3 is only one of many proteins in the viral replication complex, which includes other nsPs and host proteins. Thus, the actual antiviral mechanism of SRI-43750 may be complex and further investigation to determine whether SRI-43750 can block the ADP-ribosylhydrolase activity of nsP3MD is warranted to understand the mechanism of CHIKV replication inhibition. Further development of the pyrimidone compound may ultimately lead to the identification of a highly potent pyrimidone compound at the nanomolar range that can significantly inhibit the ADP-ribosylhydrolase activity of nsP3MD. The compound could be used to identify the mechanism of action for pyrimidone series and determine whether it is acting through inhibiting the ADP-ribosylhydrolase enzymatic activity of nsP3MD or another activity of nsP3.

### *In silico* compound design of pan-antiviral compounds targeting viral macrodomains

**Fig 7:**
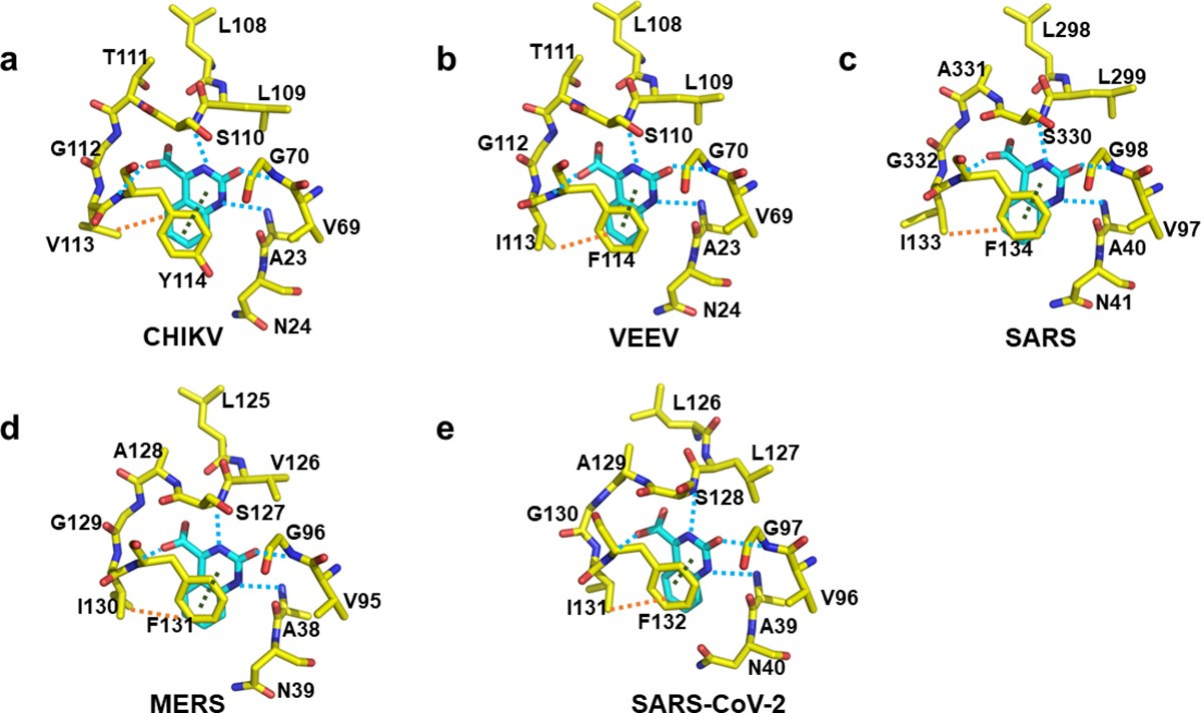
The structure of CHIKV nsP3MD-SRI-43750 and the docking results of SRI-43750 bound to macrodomains from VEEV, SARS, MERS and SARS-CoV-2. a) Co-crystal structure of CHIKV nsPMD-SRI-43750 (PDB ID: 6W8M). b) Docking model of SRI-43750 in the macrodomain of VEEV (PDB ID: 3GQO) [29]. c) Docking result of SRI-43750 in the macrodomain of SARS (PDB ID: 2FAV) [53]. d) Docking model of SRI-43750 in the macrodomain of MERS (PDB ID: 5HOL) [54]. e) Docking model of SRI-43750 in the macrodomain of SARS-CoV-2 (PDB ID: 6W02) [53]. The conserved residues interacting with SRI-43750 in the macrodomain structures of alphaviruses and coronaviruses are highlighted in yellow sticks and SRI-43750 shown in cyan sticks in all structures. Hydrogen bonds, hydrophobic contacts, and π-π stacking are indicated by cyan, orange, and dark green dashed lines respectively.

The macrodomain is conserved among the alphaviruses (Fig 2). Interestingly, the coronavirus family also contains a macrodomain that has a structure similar to nsP3MD [52]. The unique pyrimidone compounds described above could bind to nsP3 macrodomains in other alphaviruses as well as coronaviruses since the macrodomain structures and residues contacting the 2-pyrimidone-4-carboxylate core structure are highly conserved (Fig 2). Previous studies have shown that the macrodomain is very critical for viral replication for these viral families [52]. Therefore, using a small molecule to inhibit the macrodomain activity could be a novel therapeutic approach to treat the diseases caused by infections with alphaviruses and coronaviruses alike. Our computational studies using SRI-43750 suggest that compounds with the unique pyrimidone scaffold have the potential to be developed into pan-antiviral agents for alphaviruses and coronaviruses. The docking results showed that the key interactions (the π-π stacking, the hydrogen bonding network and hydrophobic interactions) and residues responsible for the binding of the pyrimidone scaffold in the macrodomains are highly conserved throughout these viral proteins (Fig 2) probably due to the required ADP-ribose binding activity for all viral macrodomains. In detail, the pyrimidone ring of SRI-43750 can still form π-π packing with the phenyl ring of phenylalanine residues in VEEV (F114), SARS (F133), MERS (F131), and SARS-CoV-2 (F132) that are equivalent to a tyrosine (Y144) in CHIKV (Fig 7). The carboxylate group and all heteroatoms on the pyrimidone ring form a similar hydrogen-bond network with protein backbones in VEEV, SARS, MERS, and SARS-CoV-2 (Fig 7). The hydrophobic contacts with the side chain of a valine (V113) in CHIKV nsP3MD can be recaptured with *iso*leucine residues in VEEV (I113), SARS (I132), MERS (I130), and SARS-CoV-2 (I131) as well (Fig 7). Besides these interactions, the conserved asparagine residue (N24) important for viral replication in CHIKV is highly conserved in other alphaviruses and coronaviruses (N24 in VEEV, N41 in SARS, N39 in MERS, and N40 in SARS-CoV-2) (Fig 2) and is close to SRI-43750 in the docking models (Fig 7). Overall, this *in silico* comparison indicates that the scaffold reported here can be used to design novel pyrimidone inhibitors of both alphaviruses and coronaviruses. The design of specific pyrimidone inhibitors for an individual virus can be achieved by designing chemical groups to interact with the various residues which are different in the ADP-ribose binding site of each virus.

## Conclusions

Exploring the approaches of FBDD, we found a conserved pyrimidone scaffold, 2-pyrimidone-4-carboxylic acid in the nsP3MD crystal structures. One fragment from the scaffold, 2-oxo-1*H*-quinazoline-4-carboxylic acid, showed anti-CHIKV viral activity with IC_50_ of 23 μM. It is the first anti-CHIKV inhibitor identified that specifically targets the macrodomain of CHIKV nsP3. Our studies described here provide a framework to further develop the pyrimidone scaffold to an anti-CHIKV lead molecule in future. Furthermore, the computational studies show that pyrimidone-based inhibitors can also have potential pan-antiviral activity against both alphaviruses and coronaviruses due to the conserved binding pockets on the viral macrodomains.

## Supporting information

S1 Table Statistics of data collection, processing and refinement. (DOCX)

S2 Table Virtual hits purchased for the first round of co-crystal screen. (DOCX)
