# Supplementary material for: Pyrimidone inhibitors targeting Chikungunya Virus nsP3 macrodomain by fragment-based drug design
Source: PLoS One. 2021 Jan 22;16(1):e0245013. doi: 10.1371/journal.pone.0245013 (PMC7822648; doi:10.1371/journal.pone.0245013)
Supplement: S1 Table — (DOCX) [file pone.0245013.s001.docx]

| **S1 Table. Statistics of Data collection, processing and refinement.** | | | | |  |  |
| --- | --- | --- | --- | --- | --- | --- |
| Values in parentheses are for the outer shell. | | | | | | |
| **Co-crystals of nsP3MD** | ADPR | 40582 | 42616 | 42642 | 43750 | |
| **PDB ID** | 6VUQ | 6W0T | 6W7H | 6W8K | 6W8M | |
| **Data collection and process** | | | | | | |
| X-ray source | SERCAT-ID-22^a^ | SERCAT-ID-22^a^ | SERCAT-ID-22^a^ | SERCAT-ID-22^a^ | SERCAT-ID-22^a^ | |
| Wavelength (Å) | 1.0 | 1.0 | 1.0 | 1.0 | 1.0 | |
| Detector | Rayonix MX300HS | Rayonix MX300HS | Eiger 16M | Eiger 16M | Eiger 16M | |
| Space group | P31 | | | | | |
| **Cell dimensions** | | | | | | |
| a,b, c (Å) | 88.08, 88.08, 83.78 | 87.77, 87.77, 83.11 | 87.44, 87.44, 83.918 | 88.013, 88.013, 83.552 | 87.958, 87.958, 83.381 | |
| α, β, γ (°) | 90, 90, 120 | | | | | |
| Mosaicity (°) | 0.15 | 0.12 | 0.11 | 0.11 | 0.31 | |
| Resolution range (Å) | 76.29-1.64  (1.73-1.64) | 76.28-1.95  (2.0-1.95) | 75.74-2.0  (2.052-2.0) | 76.23-1.80  (1.847-1.8) | 76.18-1.75  (1.795-1.75) | |
| Total unique reflections | 88999 | 52180 | 48495 | 67119 | 72803 | |
| I/sigI (I) | 17.1 (2.7) | 14.2(2.8) | 15.4(3.1) | 13.8(2.7) | 16.6(2.5) | |
| Completeness (%) | 99 (99.7) | 98.6(98.5) | 100(99.9) | 99.2(100) | 100(100) | |
| multiplicity | 5.6(5.4) | 5.3(5.4) | 5.9(6.0) | 5.7(5.8) | 5.6(5.7) | |
| R_merge_ | 0.052(0.61) | 0.061(0.514) | 0.056(0.547) | 0.069(0.566) | 0.041(0.547) | |
| **Structure refinement** |  | | | | | |
| No. reflections, working set | 84554(6179) | 48724(3570) | 46013(3389) | 62874(4642) | 69026(5130) | |
| No. reflections, free set | 4420(312) | 2700(196) | 2467(196) | 3323(270) | 3754(290) | |
| No. of non-hydrogen atoms | 5442 | 5042 | 5286 | 5019 | 5274 | |
| R_work_/R_free_^b^ | 18.71/20.63 | 22.81/25.70 | 19.13/23.12 | 20.95/24.79 | 20.40/23.66 | |
| rms deviations |  |  |  |  |  | |
| Bond distance (Å) | 0.007 | 0.007 | 0.008 | 0.012 | 0.005 | |
| Bond angles (°) | 1.369 | 1.376 | 1.435 | 1.507 | 1.313 | |
| Average B factor (A°^2^) |  |  |  |  |  | |
| protein | 28.08 | 48.89 | 42.48 | 44.39 | 42.03 | |
| fragment | 25.63 | 54.55 | 34.18 | 35.81 | 33.75 | |
| Overall figure of merit (%) | 85.01 | 80.39 | 82.27 | 81.48 | 81.34 | |
| Ramachandran plot | | | | | | |
| Most favored regions (%) | 93.7 | 93.1 | 93.5 | 93.6 | 93.3 | |
| Additional allowed (%) | 6.3 | 6.9 | 6.5 | 6.4 | 6.7 | |

1. Data was collected at beamline ID-22 at SER-CAT located at Advanced Proton Sources, Argonne National Laboratory

b. The free R factor was calculated using a randomly selected 5% of reflections omitted from the refinement.

| **S1 Table. Statistics of Data collection, processing and refinement (cont’d)** | | | | |
| --- | --- | --- | --- | --- |
| Values in parentheses are for the outer shell. | | | | |
| **Co-crystals of nsP3MD** | 43945 | 44016 | 44019 | 44165 |
| **PDB ID** | 6W8Q | 6W8Y | 6W8Z | 6W91 |
| **Data collection and process** | | | | |
| X-ray Source | Rigaku Raxis IV++ | SERCAT-ID-22^a^ | SERCAT-ID-22^a^ | Rigaku Raxis IV++ |
| Wavelength (Å) | 1.54 | 1.0 | 1.0 | 1.54 |
| Detector | Pilatus 200K | Eiger 16M | Eiger 16M | Pilatus 200K |
| Space group | P31 | | | |
| **Cell dimensions** | | | | |
| a,b, c (Å) | 87.379, 87.379, 83.333 | 87.427, 87.427, 83.888 | 87.676, 87.676, 83.851 | 87.03, 87.03, 83.845 |
| α, β, γ (°) | 90, 90, 120 | | | |
| Mosaicity (°) | 0.6 | 0.06 | 0.08 | 0.3 |
| Resolution range (Å) | 75.68-2.35  (2.405-2.35) | 75.47-2.1  (2.155-2.1) | 75.94-1.9  (1.949-1.9) | 75.38-2.2  (2.268-2.2) |
| Total unique reflections | 29893 | 41833 | 56820 | 35519 |
| I/sigI (I) | 11.7(2.3) | 10.0 (3.3) | 14.7(2.0) | 17.4(1.8) |
| Completeness (%) | 100(100) | 99.7(99.7) | 99.3(93.2) | 99.7(97.6) |
| multiplicity | 4.1(3.7) | 6.0(6.1) | 5.6(3.5) | 1.9(1.5) |
| R_merge_ | 0.086(0.572) | 0.114(0.499) | 0.063(0.579) | 0.07(0.51) |
| **Structure refinement** | | | | |
| No. reflections, working set | 28383(2085) | 39589(2929) | 5.547(3744) | 3371(2426) |
| No. reflections, free set | 1469(118) | 2096(132) | 2887(232) | 1724(107) |
| No. of non-hydrogen atoms | 5143 | 5017 | 5126 | 5069 |
| R_work_/R_free_^b^ | 21.86/26.92 | 20.84/25.46 | 20.66/25.64 | 21.25/27.11 |
| rms deviations |  |  |  |  |
| Bond distance (Å) | 0.003 | 0.008 | 0.009 | 0.007 |
| Bond angles (°) | 1.233 | 1.504 | 1.601 | 1.395 |
| Average B factor (A°^2^) |  |  |  |  |
| protein | 44.389 | 56.44 | 59.41 | 50.91 |
| fragment | 31468 | 56.859 | 59.41 | 53.48 |
| Overall figure of merit (%) | 75.91 | 78.13 | 78.97 | 77.14 |
| Ramachandran plot | | | | |
| Most favored regions (%) | 91.6 | 90.5 | 90.9 | 92.5 |
| Additional allowed (%) | 8.4 | 9.5 | 9.1 | 7.5 |

a. Data was collected at beamline ID-22 at SER-CAT located at Advanced Proton Sources, Argonne National Laboratory

b. The free R factor was calculated using a randomly selected 5% of reflections omitted from the refinement.
