# Supplementary material for: Pyrimidone inhibitors targeting Chikungunya Virus nsP3 macrodomain by fragment-based drug design
Source: PLoS One. 2021 Jan 22;16(1):e0245013. doi: 10.1371/journal.pone.0245013 (PMC7822648; doi:10.1371/journal.pone.0245013)
Supplement: S2 Table — (DOCX) [file pone.0245013.s002.docx]

**S2 Table. Virtual hits purchased for the first round of co-crystal screen**

| Structure | VS Score (kcal/mol) | Vendor and ID | Molecular Weight |
| --- | --- | --- | --- |
|  | -9.9 | Life Chemicals F2145-0404 | 196.21 |
|  | -9.9 | Chembridge 9116673 | 278.35 |
|  | -9.8 | Chembridge 9221205 | 271.32 |
|  | -9.6 | Chembridge 7984549 | 259.30 |
|  | -9.5 | Life Chemicals F3261-0132 | 264.34 |
|  | -9.3 | Chembridge 6204089 | 246.27 |
|  | -9.2 | Chembridge 9267768 | 261.26 |
|  | -9.2 | Chembridge 5814513 | 277.71 |
|  | -9.2 | Life Chemicals F0699-0003 | 228.25 |
|  | -9.1 | Chembridge 9114158 | 279.30 |

**S2 Table. Virtual hits purchased for the first round of co-crystal screen (cont’d)**

| Structure | VS Score (kcal/mol) | Vendor and ID | Molecular Weight |
| --- | --- | --- | --- |
|  | -9.0 | Chembridge 7932922 | 252.28 |
|  | -8.9 | Chembridge 9262180 | 279.25 |
|  | -8.9 | Enamine Z1416166781 | 179.18 |
|  | -8.9 | Enamine Z1626921209 | 241.29 |
|  | -8.6 | Maybridge CC24201 | 199.21 |
|  | -8.5 | Chembridge 9110708 | 285.32 |
|  | -8.4 | Life Chemicals F1957-0053 | 163.14 |
|  | -8.3 | Life Chemicals F0866-0142 | 295.30 |
|  | -8.3 | Enamine Z424777974 | 206.20 |
|  | -8.2 | Life Chemicals F1926-0019 | 188.19 |

**S2 Table. Virtual hits purchased for the first round of co-crystal screen (cont’d)**

| Structure | VS Score (kcal/mol) | Vendor and ID | Molecular Weight |
| --- | --- | --- | --- |
|  | -8.1 | Enamine Z1198163863 | 163.14 |
|  | -8.0 | Enamine Z197490120 | 235.22 |
|  | -7.9 | Chembridge 4020493 | 203.20 |
|  | -7.9 | Chembridge 9034937 | 244.23 |
|  | -7.8 | Chembridge 9193331 | 237.26 |
|  | -7.8 | Enamine Z415638506 | 193.18 |
|  | -7.8 | Chembridge 4032314 | 210.62 |
|  | -7.7 | Chembridge 4402022 | 205.21 |
|  | -7.7 | Chembridge 4029443 | 209.18 |
|  | -7.5 | Enamine Z25842930 | 289.38 |

**S2 Table. Virtual hits purchased for the first round of co-crystal screen (cont’d)**

| Structure | VS Score (kcal/mol) | Vendor and ID | Molecular Weight |
| --- | --- | --- | --- |
|  | -7.3 | Chembridge 4032335 | 190.20 |
|  | -7.2 | Life Chemicals F1901-0136 | 257.27 |
|  | -6.5 | Enamine Z56870750 | 229.24 |
|  | -6.2 | Enamine Z1509397284 | 202.22 |
|  | -6.2 | Chembridge 5211823 | 241.25 |
|  | -6.1 | Enamine Z31603048 | 244.25 |
|  | -5.4 | Enamine Z1691078292 | 233.27 |
|  | -5.3 | Chembridge 6629058 | 203.20 |
|  | -5.3 | Enamine Z316121804 | 218.21 |
|  | -5.1 | Enamine Z1269130394 | 204.11 |
